# Long-read metagenomics and methylation-based binning support the discovery of antibiotic resistance gene–host associations in complex communities

**DOI:** 10.1186/s13059-026-04200-0

**Published:** 2026-07-13

**Authors:** Melina Markkanen, Heidi Putkuri, Dovydas Kičiatovas, Ville Mustonen, Marko Virta, Antti Karkman

**Affiliations:** 1 Department of Microbiology, University of Helsinki, Helsinki, Finland; 2 Multidisciplinary Center of Excellence in Antimicrobial Resistance Research, University of Helsinki, Helsinki, Finland; 3 Department of Computer Science, Department of Organismal and Evolutionary Biology, University of Helsinki, Helsinki, Finland

**Keywords:** Antibiotic resistance, DNA methylation, Long-read metagenomic sequencing, Wastewater, P*dif*-modules

## Abstract

**Background:**

Antibiotic resistance genes (ARGs) circulating among clinically relevant bacteria pose serious challenges to public health. Given the ancient and environmental bacterial origins of ARGs, a better understanding of the carriers of ARGs beyond the clinically most relevant species is urgently needed for longer-term resistance monitoring and intervention measures. While the risks of emerging ARGs from environmental sources have been recognized, the identification bottlenecks stem from the limitations of shotgun metagenomic sequencing and bioinformatic methods.

**Results:**

We use long-read metagenomic sequencing and bacteria-specific methylation profiles to re-establish the links between established (well-described) or latent (absent in databases) ARGs and their bacterial and genetic contexts in wastewater. We analyze base modification data produced by PacBio SMRT sequencing using an in-house pipeline utilizing position weight matrices and UMAP visualizations, which we validate by a synthetic community with known bacterial composition. Our analysis reveals several previously unreported ARGs and ARG-host linkages in wastewater. For instance, we find that *Arcobacter*, a key wastewater associated taxon and emerging pathogen, carries a latent beta-lactamase gene with high predicted mobility potential. Of the other understudied beta-lactamases, we describe *bla*MCA within p*dif-*modules across highly varying contexts suggesting its recent acquisition events. Additionally, we uncover the wastewater resident taxa mediated carriage of clinically important ARGs.

**Conclusions:**

By linking ARGs to their wider genetic contexts and hosts, our findings shed light on the previously unrecognized carriers of resistance genes in wastewater. The presented approach provides a valuable methodology for early identification of newly arising ARGs and their hosts.

**Supplementary Information:**

The online version contains supplementary material available at https://doi.org/10.1186/s13059-026-04200-0.

## Background

The introduction of antibiotics into therapeutic practice represents a pivotal milestone in the advancement of medicine [1]. However, the simultaneous increase in emergence and circulation of antibiotic resistance genes (ARGs) has severely diminished the efficacy of the antibiotic drugs available in human and veterinary medicine, challenging the entire health care system. ARG mobilization is enabled by horizontally transferred mobile genetic elements (MGEs) and their adjacent genes. The resistance features carried by the most common opportunistic pathogens are relatively well described owing to their immediate clinical relevance, but also due to practical reasons, as many of these bacteria are cultivable. However, given the complexity of the resistance gene dynamics including non-pathogenic bacteria, and rapidly evolving resistance situation, a better understanding of the ARG mobility mechanisms and ARG carriers beyond the cultivable clinical strains is urgently needed [2].

The study of environmental resistomes enables the identification of both established and latent ARGs–that is, those that are well-characterized in databases and those that are largely unexplored–regardless of the cultivability of their host bacteria [3]. For instance, a wide range of established and latent beta-lactamase genes are highly frequent among environmental bacteria, especially within wastewater communities [3–5]. Indeed, owing to the high diversity of bacteria from human and environmental sources, persisting under diverse stressors [6], wastewater has been identified as one of the key environments regarding the presence of latent ARGs [3] with elevated risk of becoming clinically problematic if mobilized into relevant opportunistic pathogen hosts [3, 7]. The transition from a non-mobile context in environmental bacteria to clinically relevant ARGs circulating among human pathogens contains multiple gene decontextualization and host transition events by intermediate hosts [2, 7]. Unfortunately, considering the prevailing conditions of the post-antibiotic era, this transfer may be irreversible [8]. Several environmental origin bacteria and intermediate hosts (or recent hosts) of the current clinical ARGs have been resolved [4, 9–13]. Although due to detection difficulties [2], this is typically done retrospectively. Knowledge about the intermediate ARG carriers is particularly important, as they may supply the foundational genetic material for the emergence of clinically relevant resistant bacteria [14].

Ranking ARGs in environmental resistomes by their impact on human health is inherently difficult and remains debatable [15, 16]. The risk‑ranking schemes that have given prominence to mobile ARGs that are already present in pathogens [16, 17] have been challenged by more farsighted view, which define novel mobile resistance determinants arising from environmental microbiomes, as the most critical contributors to the current rapidly developing resistance problem [15]. These schemes aim for early capture of emerging resistance threats by assessing the likelihood of ARG transmission from environmental species to clinically relevant bacteria and the severity of such an event [15], using ARG mobility as the proxy for this [2]. However, the inability of shotgun metagenomic sequencing [18] and assembly [19] in linking ARGs, especially those encoded by MGEs, to their host bacteria has substantially limited our understanding of the environmental bacterial ARG hosts [2, 15]. Metagenomic assembly with long-reads may allow simultaneous identification of ARGs and their near genetic context, but their hosts remain unresolved, especially in highly complex communities, such as influent wastewater [2]. Current metagenomic binning algorithms, which rely on sequence composition and coverage, often overlook ARGs located on MGEs, for example, failing to link plasmids to their host bacteria due to differential coverage [20, 21]. This remains one of the most significant challenges in the entire field of microbial metagenomics and antibiotic resistance research, as plasmids carry vital adaptation-increasing and evolution-driving features [21, 22]. Additionally, binning algorithms are confused by the presence of multiple strains of one species, biased toward the high-abundance taxa while ignoring the genomes of rare species, which the ARG-carrying bacteria commonly are [3, 23]. Consequently, these biases leave us with persistent uncertainties regarding the role of environmental lineages on the ARG carriage and spread.

Bacterial DNA methylation signals are base modifications mediated by target sequence motif-specific methyltransferase enzymes, often acting as part of the restriction–modification systems [21]. As the main epigenetic modification mechanism of bacterial genomes, methylation has roles in various regulatory functions, gene expression [24, 25], and genome defense against phages [26] by enabling the distinction between own and foreign DNA [25]. Due to the unique set of methylation-mediating enzymes and their unique target motifs, all sequence material of a bacterial strain (chromosomes and plasmids) is expected to show strain-specific methylation profiles [21], allowing their bioinformatic reconnection into genome bins [11, 21]. So far, the bacterial methylation detection with the latest technology by PacBio and Nanopore, allowing direct methylation detection without pretreatment [25], has been focused on isolates, while fewer analysis tools and workflows are available for metagenomic datasets, except those with low bacterial complexity [21, 27–30]. For instance, the computational demands required for a comprehensive analysis of highly complex wastewater microbial communities with the MultiMotifMaker algorithm [31] extend beyond what is practically achievable. Tools similar to Nanomotif [20], which is specifically designed for Nanopore data, are greatly needed to take advantage of the kinetic data produced by PacBio SMRT sequencing.

Although a comprehensive range of tools exists for detecting resistance gene features in microbiome data, approaches for resolving ARG-host associations in microbial communities are still lacking. In this study, we focused on revealing the genetic and bacterial contexts of ARGs–particularly those located near mobile genetic elements or representing computationally predicted latent ARGs, undetected by other methods–by the application of long-read metagenomic sequencing and DNA methylation analysis. The presented pipeline uses position weight matrices (PWMs) to summarize the contig-wise PacBio base modification data. Uniform Manifold Approximation and Projection (UMAP) is applied to cluster metagenomic contigs based on their methylation profiles, facilitating the generation of bins of connected contigs (or “genome bins” hereafter) and thereby reveal the ARG-carrying bacteria present in wastewater. A synthetic community with known bacterial composition was used to validate our methodology. In contrast to individual metagenomic contigs, the ability to form genome bins, even in incomplete form, provides sufficient genomic context to support predictions of ARG mobility and host species assignment. We focused on carriers of both established and latent ARGs in influent, effluent, and dried sludge to better understand environmental bacteria diversity linked to ARG carriage, and to identify previously uncharacterized, potentially further transferable ARGs.

## Results

### Position Weight Matrix (PWM) -based method for linking contigs by their shared methylation signatures

**Fig. 1:**
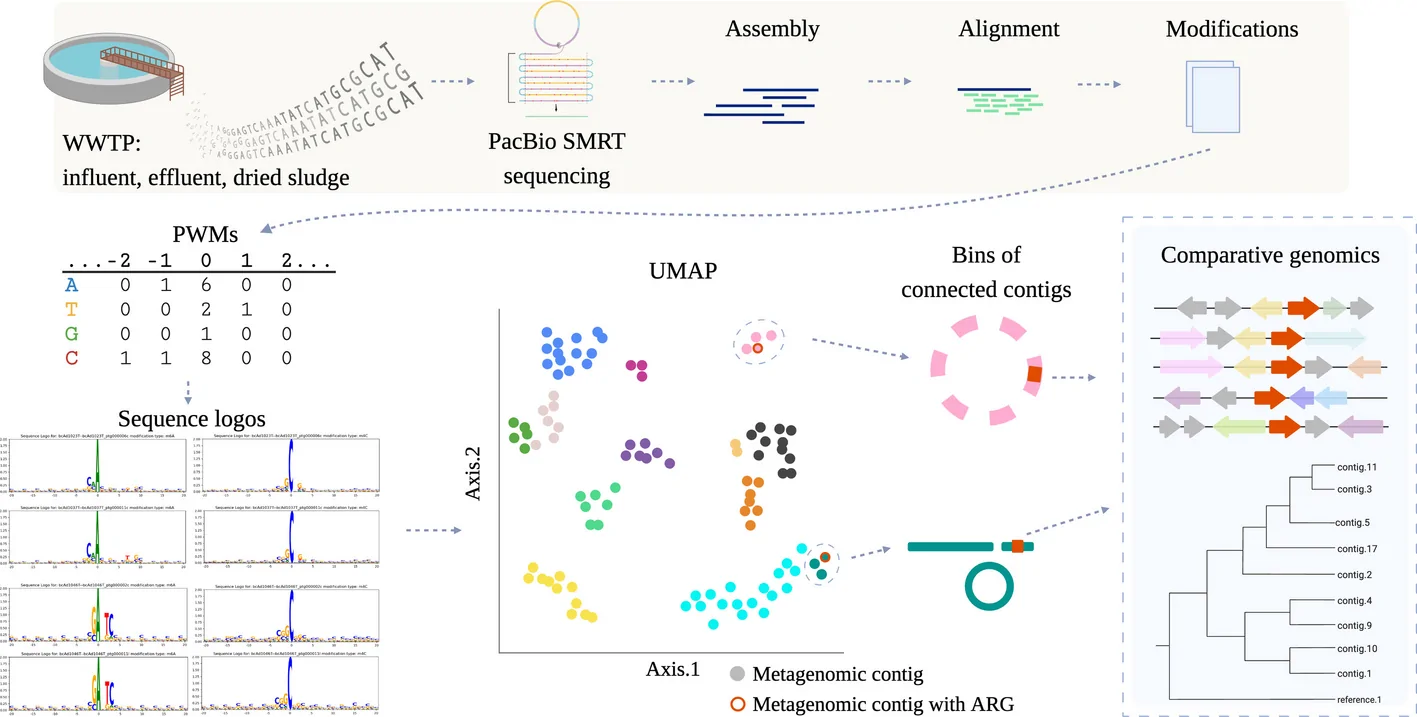
Schematic representation of the key steps in the workflow. Before the analysis of the wastewater samples, the workflow (until the genome bin formation step) was applied on a synthetic community with known bacterial composition as a method validation procedure. Figure created with BioRender.com

**Fig. 2:**
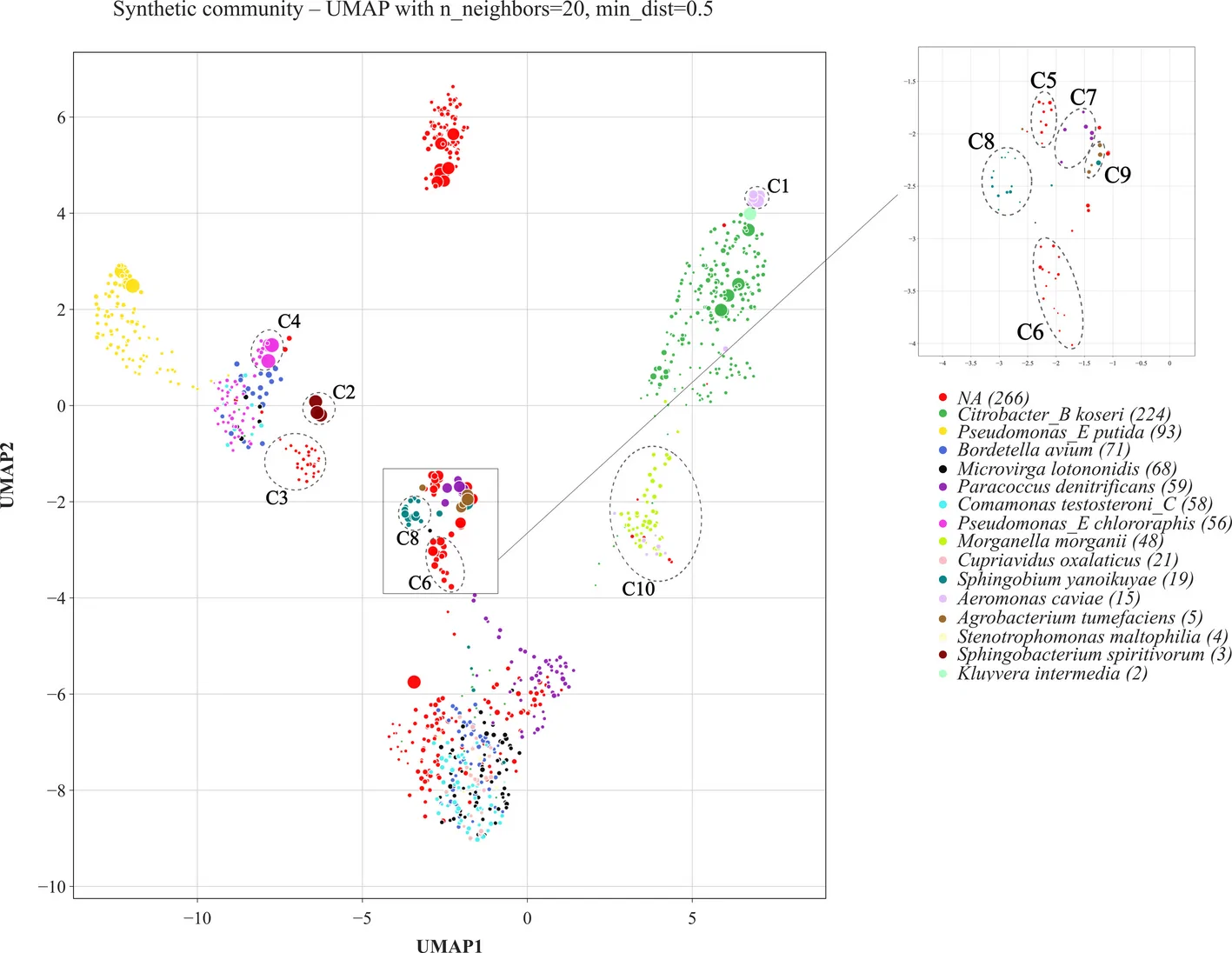
UMAP of the synthetic community methylomes. The a priori known taxonomical identities of the metagenomic contigs are color-coded based on species-level taxa. Numbers after species names indicate the number of contigs included in the visualization. Data point size denotes the square root transformed contig length. Clusters C1-C10 were defined by visual inspection and used for genome binning (Additional file 1: Table S2)

In the absence of suitable tools for methylome analysis of PacBio SMRT sequencing data from complex microbial community samples, such as wastewater, a new workflow was established here (Fig. 1). In our approach, the per-contig base modifications were converted into position weight matrices (PWMs), allowing clustering according to their methylation profile by UMAP. Distinctly clustered contigs were used to form genome bins, based on the assumption that all genetic material within a cell, methylated by a shared set of MTases, exhibits a uniform bacteria-specific signal [21]. We validated our workflow with a synthetic community of known bacterial composition, of which whole genome sequence (WGS) data were available for half of the community members (Fig. 2, Additional file 1: Table S1).

PacBio SMRT sequencing of nine wastewater samples resulted in an average of 7 345 157 reads and 65 643 contigs per sample (Table 1). Corresponding values for the five synthetic community samples were 25 0865 and 398 (Table 1). Contigs with more than 50 base modifications were included in the methylation analysis covering on average, 63.0% of all contigs in the synthetic community samples and 26.2% in the wastewater samples. Visual inspection of the ± 20 base sequence window surrounding the modified bases by sequence logos consistently showed that position 0 corresponded to A for m6A and C for m4C, as expected (Additional file 2: Fig. S1).

**Table 1:** Sequencing and metagenomic assembly statistics and the number of contigs included in the methylation-based binning analyses (contigs with > 50 base modifications)

Data set	Sample	Reads	Total contigs	Contigs with > 50 modifications	Contigs after secondary filtering^a^	Contigs retained for clustering (%)^b^
**Synthetic community**						
	bcAd1023	209887	305	182		59.7
	bcAd1037	272936	336	204		60.7
	bcAd1039	303076	713	470		65.9
	bcAd1046	163417	263	128		48.7
	bcAd1063	305009	373	298		79.9
**Wastewater community**						
	INF1	6963058	65422	34630	9657	14.8
	INF2	6864058	62366	33542	9950	16.0
	INF3	7080557	62921	29835	9778	15.5
	EFF1	7706135	60537	31940	16797	27.7
	EFF2	6981303	59860	29253	14976	25.0
	EFF3	6658177	54991	28927	17524	31.9
	SLU1	7587277	74572	38510	24736	33.2
	SLU2	8285626	76311	41305	27251	35.7
	SLU3	7980218	7311	40449	26669	36.1

^a^Number of contigs retained after excluding the contigs of miscellaneous clusters on UMAP visualization

^b^Proportion between all contigs with > 50 modifications and total contigs

Metagenomic contigs from synthetic communities were visualized by UMAP, where they clustered based on their methylation profiles encoded as PWMs, which could be used to recover contig relationships lost during shotgun sequencing. To validate formed clusters, completion and redundancy values, computed by CheckM2 (v 1.0.1) [32] of the resulting genome bins were examined (Additional file 1: Table S2). Five high-quality genome bins (completion > 90%, redundancy < 5%) representing three species were recovered, most containing both chromosomal and plasmid elements; four medium-quality (completion > 50%, redundancy < 10%) and six partial genomes (10–50%, < 10%) represented four and five species, respectively. The latter two groups included five genome bins from species lacking WGS data for taxonomical validation (Fig. 2, Additional file 1: Table S1, Additional file 1: Table S2). The partial genome bins and the differences in how different species were covered by genome bins was most likely due to the application of relatively stringent contig inclusion criteria (contigs with > 50 modifications) resulting in fewer contigs available for binning (Table 1) as well as differences in the species relative abundances in the communities [33]. Additionally, some contigs lacked distinct methylation signatures either due technical limitations, such as sensitivity, or biological constrains like cell state-dependent methylation variation [34]. However, as this miscellaneous group of contigs was clearly separate from the more defined, species-specific distinct clusters (Fig. 2), the false positives from these contigs were unlikely. Altogether, based on the observations on the synthetic community, we concluded that our approach was able to reconnect metagenomic contigs into species-wise genome bins and could therefore be applied to wastewater data to establish ARG-host linkages.

### General overview of the wastewater genome bins with ARGs

As expected, not all contigs originating from wastewater data produced distinct methylation patterns (Additional file 2: Fig. S2) and those were excluded from further analysis (“Contigs after secondary filtering” in Table 1). After UMAP clustering and genome bin quality assessment, we obtained, 35 genome bins with established and 53 with latent ARGs (Table 2). The average completion and redundancy values were 55.40 and 2.17, respectively, and the GC-content of the genome bins matched their closest reference genomes in GTDB (Additional file 1: Table S3). On average, the genome bins were composed of 12 contigs (range from 2 to 44) (Additional file 1: Table S3). Of all genome bins, 31 were obtained from influent, 15 from effluent, and 42 from dried sludge (Table 2, Additional file 1: Table S3). These numbers represented only small proportion of the established and latent ARGs detected in all assembled contigs and even among those retained after secondary contig filtering (Table 2). Hence, due to the non-quantitative nature of the approach, the resulting genome bins cannot be considered a quantitative measure of ARG-encoding bacteria in the samples.

**Table 2:** Methylation-based genome bins encoding latent and established ARGs

Sample	Sample type	Genome bins with established ARGs	Total established ARGs after secondary contig filtering^**a**^	Total established ARGs	Genome bins with latent ARGs	Total latent ARGs after secondary contig filtering^a^	Total latent ARGs
INF1	influent	2	83	796	6	120	526
INF2	influent	4	86	565	7	133	507
INF3	influent	5	56	398	7	128	397
EFF1	effluent	1	14	53	2	47	215
EFF2	effluent	1	11	39	6	57	213
EFF3	effluent	1	5	16	4	76	126
SLU1	dried sludge	7	34	136	5	121	316
SLU2	dried sludge	9	51	136	7	169	343
SLU3	dried sludge	5	54	155	9	166	351
Total		35	349	2294	53	1017	2994

^a^Number of ARGs after excluding the contigs of miscellaneous clusters on UMAP visualization

Given the pivotal role of plasmid-encoded ARGs for the antibiotic resistance problem, we were particularly interested in linking plasmids to their hosts. Although we could not link any ARG-encoding plasmids to their hosts, we could find linkages between non-ARG-carrying plasmids and their host genome bins in several cases (Additional file 1: Table S4). For instance, two to five plasmids were linked to *Acinetobacter* sp. in three different genome bins from influent (bins INF1-C3f, INF1-C6f, and INF2-C2f) (Additional file 1: Table S4). Single plasmids were identified, for *Aquabacterium_A* sp. (bin EFF2-C1f) and *Aeromonas media* (bin INF1-C4) (Additional file 1: Table S4). BLAST searches against NCBI matched these contigs to plasmid sequences (data not shown), confirming our approach’s ability to connect chromosomal and plasmid sequences, at least non-mobile, lineage specific plasmids.

*bla*OXA genes carrying *Aeromonas* sp. were most commonly identified as established ARG carriers in influent, while Bacteroidales order bacteria carrying *erm*(F) genes were descriptive for dried sludge. The fewer genome bins with established ARGs obtained from effluent were mainly Burkholderia order bacteria carrying various established ARGs. Regarding latent ARGs, beta-lactamases of classes A, B 1–3, C, and D, most notably of the latter, were identified in more diverse hosts, including bacteria belonging to 16 different orders such as Burkholderiales, Bacteroidales, Pseudomonadales, Chitinophagales, Campylobacterales, and Christensenellales, to name the most common groups. In general, the latent ARG host species included more members of the uncultivated bacterial lineages compared to the identified carriers of established ARGs. We next focused on a couple of the most interesting ARG-host linkages (Additional file 1: Table S3).

### Subset of the latent class D beta-lactamase variants linked to *Arcobacter* exhibited high mobility potential

**Fig. 3:**
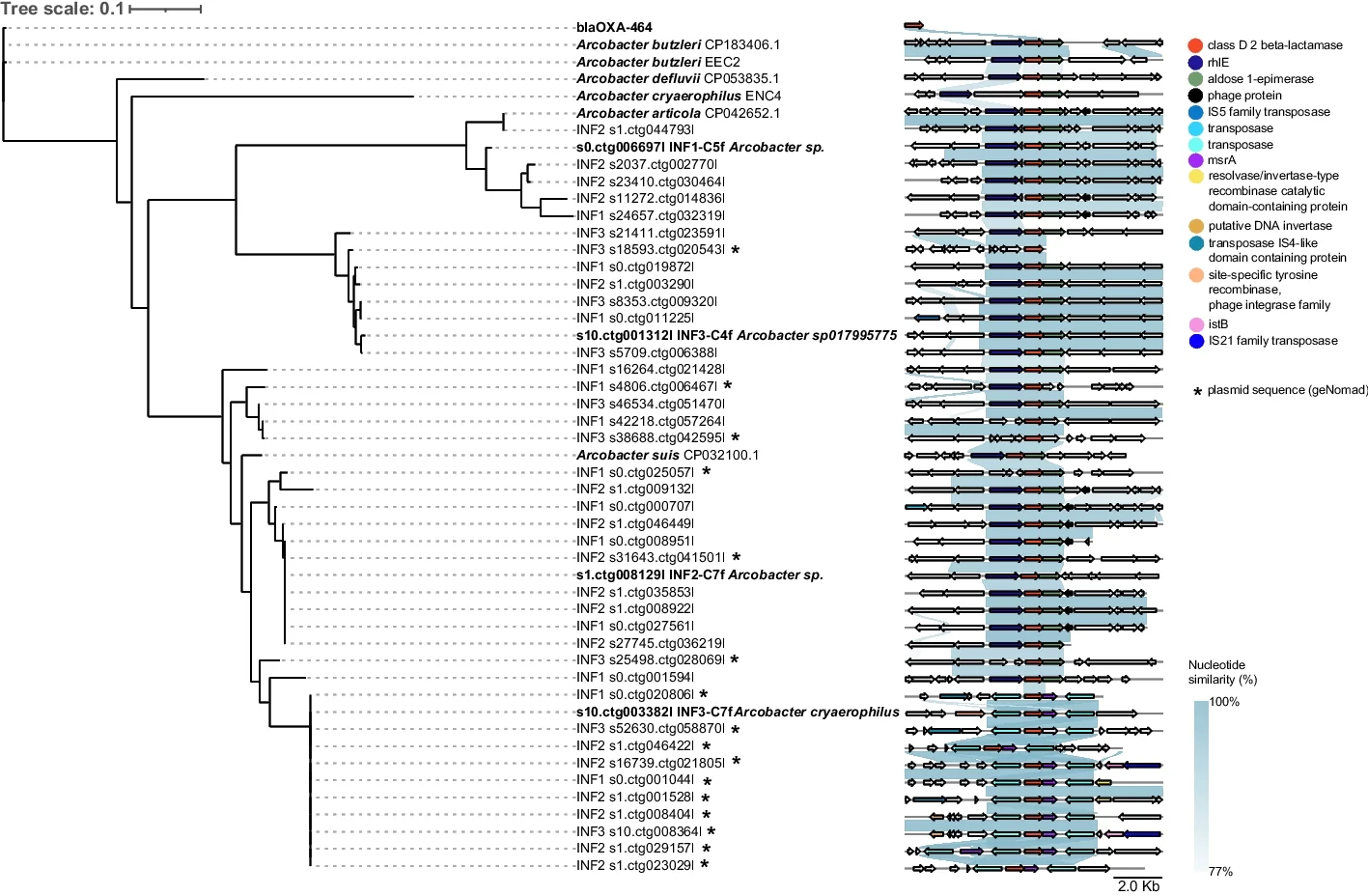
Phylogenetic tree constructed from the latent class D beta-lactamase gene (highlighted in red) and visualized alongside its genetic context within the genome bins of *Arcobacter* species resolved by methylation-based binning (bolded text) and unbinned sequences. The nucleotide similarity (%) between the sequences is shown by blue shading. Flanking regions of 5 Kb upstream and downstream of the class D beta-lactamase were included in the genetic context visualization. Reference sequences showing varying similarity to the latent gene (*Arcobacter butzleri* (CP183406.1, and EEC2), *Arcobacter defluvii* (CP053835.1), *Arcobacter cryaerophilus* (ENC4), *Arcobacter articola* (CP042652.1), *Arcobacter suis* (CP032100.1)) were included in the comparative genomics

*Arcobacter* species of the Campylobacterota phylum were one of the major bacterial groups detected in influent wastewater (Additional file 2: Fig. S3). Genome bins assigned as *Arcobacter* spp. and hosting latent class D beta-lactamase genes were recovered from each of the influent samples (bins INF1-C5f, INF2-C7f, INF3-C4f, and INF3-C7f, Additional file 1: Table S3, Additional file 1: Table S5). They showed completeness and redundancy values ranging from 21.16% to 31.69% and 0.03% to 0.8%, respectively, and were assigned as *Arcobacter* sp., *Arcobacter* sp., *Arcobacter sp017995775*, and *Arcobacter cryaerophilus A* (Additional file 1: Table S5). (The previously distinct genera *Arcobacter* and *Aliarcobacter* are referred here as a single genus *Arcobacter* according to the latest update on nomenclature and taxonomical classification [35]). The class D beta-lactamases carried by each of the four genome bins formed distinct branches within phylogenetic tree, also primarily differing from those of the reference sequences (Fig. 3).

Despite differences in the beta-lactamase gene sequences, their immediate flanking region showed marked conservation across most hosts and branches of the phylogenetic tree (Fig. 3). This conserved region consisted of genes *rhlE* and aldose 1-epimerase (mutarotase), encoding core functions related to RNA [36] and carbohydrate metabolism [37], supporting a non-mobile context of those beta-lactamases (Fig. 3). As a notable exception, this conserved region was absent in one of the tree branches that was represented by the genome bin assigned as *A. cryarophilus* (bin INF3-C7f) (Fig. 3). Instead, the latent beta-lactamase, along with the *msrA* gene [38], was flanked by transposase genes on both sides (Fig. 3). The plasmid prediction results suggested that all sequences of this branch, expect the *A. cryarophilus* genome bin itself, were plasmid-borne, implying higher mobility potential of these sequences, in contrast to those within conserved genetic contexts (Fig. 3, Additional file 1: Table S5). Additionally, IS21 family transposases were detected in some of these contexts within this branch, but not among the other branches (Fig. 3).

### *bla*MCA genes are found in p*dif*-modules across diverse genetic contexts including the chromosomal regions associated to *Acinetobacter* species

**Fig. 4:**
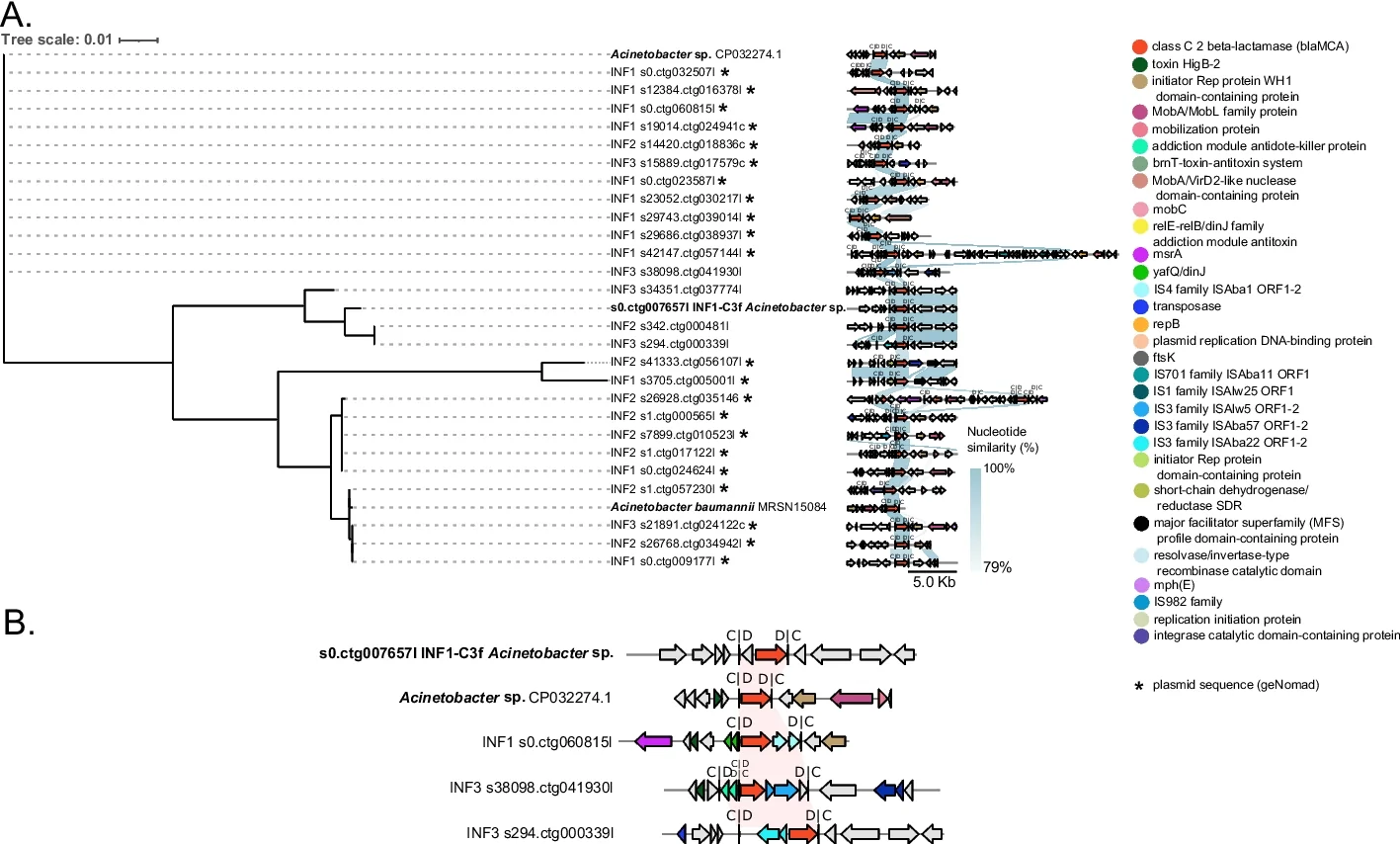
**A** Phylogenetic tree constructed from the *bla*MCA gene (highlighted in red) and visualized alongside its genetic context (5 Kb upstream and downstream) within the genome bin of *Acinetobacter* sp. and unbinned contigs. *Acinetobacter baumannii* (MRSN15084) and *Acinetobacter* sp. (CP032274.1) were included as references. **B** Gene arrangement of the p*dif*-modules containing IS3 or IS4 family transposases alongside the *bla*MCA gene. *Acinetobacter* sp. genome bin (INF1-C3f) and *Acinetobacter* sp. (CP032274.1) sequences are included in the figure for comparison

Methylation analysis enabled the characterization of high-quality (99.97% completeness and 3.43% redundancy) genome bin (bin INF1-C3f) carrying class C beta-lactamase gene *bla*MCA that was assigned as *Acinetobacter* sp. (Fig. 4A, Additional file 1: Table S3, Additional file 1: Table S6). Previous reports on *bla*MCA detection are scarce [39], possibly due to the absence of this gene in most ARG databases. Although detected here only as a latent gene by fARGene tool, which is designed to exclude established ARGs along the analysis [40], it is listed in the AMRFinderPlus database as *bla*MCA [41], a designation we adopt here.

One major branch was observed in the phylogenetic tree built on *bla*MCA gene sequences from influent wastewater (Fig. 4A). This included the reference sequence *Acinetobacter* sp. (CP032274.1) and 12 unbinned contigs that were predicted as plasmid sequences. They all encoded identical ARG sequences within highly variable genetic contexts (Fig. 4A, Additional file 1: Table S6). In contrast, the *bla*MCA gene carried by *Acinetobacter* sp. genome bin (bin INF1-C3f) formed a distinct branch and showed 92% sequence similarity (1122/1215 bp) with both references (MRSN15084 and CP032274.1) (Fig. 4A, Additional file 1: Table S6). The absence of plasmid signals together with the conserved gene arrangement across the sequences of this branch suggested a chromosomal context (Fig. 4A, Additional file 1: Table S6).

Common to all sequences was the presence of p*dif*-sites (Fig. 4, Additional file 1: Table S6, Additional file 1: Table S7). More specifically, in most cases, these C|D and D|C-oriented site-specific recombination sites were detected immediately bordering the *bla*MCA gene, forming a *bla*MCA-p*dif*-module, which was, in a few cases, present multiple times along the investigated sequence region (Fig. 4A). Despite the ancient origin of plasmid *dif* (p*dif*) recombination sites and p*dif*-module trafficking [42], this mechanism is thought to have gained additional functionality through ARG mobilization during the post-antibiotic era [43]. In three instances, different IS3 or IS4 family transposase genes were found to be co-occurring with *bla*MCA in the same p*dif*-modules (Fig. 4B). Overall, p*dif*-sites were frequently found flanking, not only *bla*MCA, but also other genes within the studied regions, likely contributing to the genetic variation observed across the studied sequences (Fig. 4).

### Gene *erm*(F) is carried by multiple different environmental Bacteroidales species in the dried sludge

**Fig. 5:**
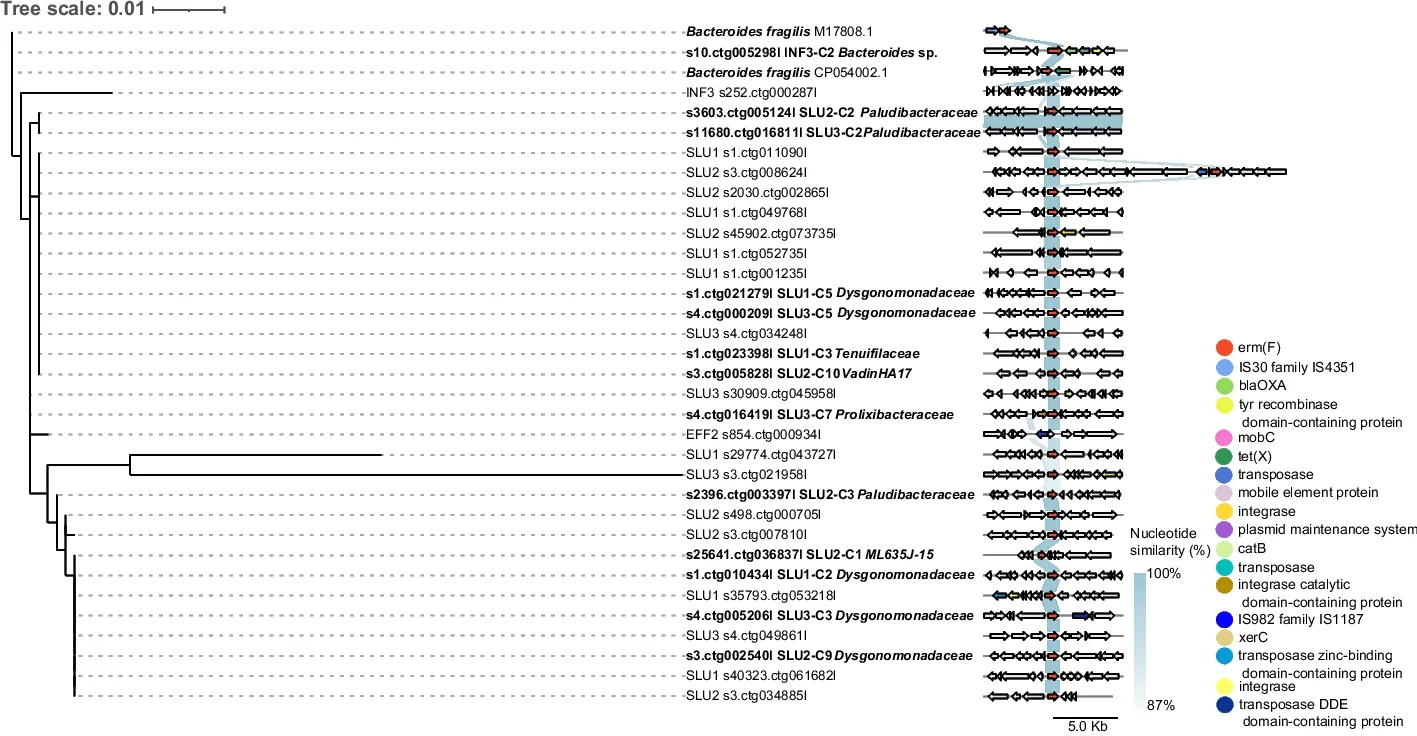
Phylogenetic tree constructed from the *erm*(F) genes (highlighted in red) and visualized alongside its genetic contexts (5 Kb upstream and downstream) identified in Bacteroidales genome bins resolved by methylation-based binning (bolded text) and unbinned contigs. *Bacteroides fragilis* (M17808.1) and *B. fragilis* (CP054002.1) encoding *erm*(F) are included as references. Family-level taxonomical identity of the genome bins is shown in bolded text

The macrolide-lincosamide-streptogramin B (MLSB) resistance gene *erm*(F) was the most frequently detected established resistance gene among all genome bins (Fig. 5, Additional file 1: Table S3, Additional file 1: Table S8). The 13 visualized *erm*(F) encoding genome bins were primarily recovered from sludge samples (Fig. 5, Additional file 1: Table S8). The completeness and redundancy values of the genome bins ranged from 17.49% to 94.92% and 0.02% to 3.84%. Altogether, they represented 7 different bacterial families within the Bacteroidales order (Additional file 1: Table S3). Of these, only one was assigned to a human gut-associated *Bacteroidetes* sp. (*Bacteroidaceae* family), and this genome bin was the only one recovered from influent (Additional file 2: Fig. S4). Instead, the taxonomical assignments of the remaining genome bins from sludge (Additional file 2: Fig. S4) represented environmental lineages of Bacteroidales, including families *Dysgonomonadaceae, Paludibacteraceae, Tenuifilaceae, Prolixibacteraceae, VadinHA17,* and *ML635J-15* (Fig. 5, Additional file 1: Table S8). These findings aligned with the significant differences in taxonomical compositions (Additional file 2: Fig. S5) and the Bacteroidota phylum relative abundances throughout the wastewater treatment process (Additional file 2: Fig. S3, Additional file 2: Fig. S6): While the human gut-associated *Bacteroidaceae* family clearly dominated in influent, the Bacteroidales taxonomic profile was markedly different in the sludge (Additional file 2: Fig. S6). There, several different Bacteroidales families present at high relative abundances, including those that were identified as *erm*(F) hosts (Fig. 5, Additional file 2: Fig. S6)*.*

Phylogenetic analysis revealed that the identified *erm*(F) genes were highly similar despite their variable genetic contexts (Fig. 5). Most of the genes shared 99–100% sequence identity with the reference *erm*(F) gene (*Bacteroides fragilis* (CP054002.1 and M17808.1), whereas the two most divergent showed 95% and 92% similarity (Fig. 5). The high plasticity of *erm*(F) flanking regions was highlighted by the description of clearly distinct gene arrangements, even between genome bins assigned to the same species of *Seramator thermalis* (bins SLU1-C5, SLU1-C2, SLU3-C3) (Fig. 5, Additional file 1: Table S8). While individual gene mobility associated elements were recognized near *erm*(F) in some sequences, no particular MGEs explaining the highly diverse *erm*(F) contexts by gene acquisition events were identified among the environmental Bacteroidales.

Additional previously unrecognized host species of well-known integron-associated ARG variants were described. The extended-spectrum beta-lactamase (ESBL) gene *bla*OXA-129 was identified in *Simplicispira* sp. genome bins from two separate influent samples (Additional file 2: Fig. S7, Additional file 1: Table S3, Additional file 1: Table S9). Similarly, two genome bins carrying sulfonamide resistance gene variant *sul1-9* within clinical class 1 integron gene cassette and assigned as *JAUCQB01* sp. of Planctomycetota phylum were recovered from separate sludge samples (Additional file 1: Table S3, Additional file 1: Table S10, Additional file 2: Fig. S8).

## Discussion

Although resistance determinants sourced from environmental microbiomes, particularly ARGs showing potential for gene mobility, have been deemed as critical contributors to the current rapidly developing resistance problem, methodological limitations have impaired their characterization [2, 15]. While a wide variety of ARGs are being identified in diverse environments, the crucial information regarding their genetic and bacterial contexts is often missing [15, 16]. instead, in many metagenomic studies, the highest-abundance taxa are often reported separately, although they rarely represent the ARG carriers in those communities [3, 23]. By leveraging a novel methylation data analysis pipeline and long-read metagenomic sequencing, this study reconstructs metagenomic contigs from complex communities, enabling the identification of previously undescribed bacterial mediators of ARG carriage in wastewater. While we uncovered only a small subset of the ARG-host linkages due to current technical limitations in methylation detection, we expect this to improve with future technological advances.

### Uncovering the bacterial and genetic contexts of latent and potentially mobile beta-lactamases

Our results pointed to *Arcobacter cryaerophilus* as the host of the latent class D beta-lactamase gene that was embedded in a composite transposon in a similar orientation as reported before for other ARGs [44–46]. Additional indications of putative gene mobility, such as plasmid context and adjacent IS21 family transposases [47], found in subset of the studied sequences could suggest increased propensity for horizontal transfer of this gene. Given that *A. cryaerophilus* is one of the *Arcobacter* species most commonly isolated in clinical cases [48], our results could possibly be indicative of ARG accumulation into clinically relevant lineages of *Arcobacter*. The remaining class D beta-lactamase genes described here in *A. sp017995775* and unidentified species of *Arcobacter* represented likely chromosomal, non-mobile variants of this gene. Still, they were dissimilar to the closest established ARG variant, *bla*OXA-464, which has been previously reported from wastewater [49] in *Arcobacter* sp. [50]. This underscored the largely uncharacterized diversity of this bacterial group [51].

As a dominant taxon and key denitrifier in wastewater [52, 53], *Arcobacter* species have been suggested to contribute to the ARG load in influent. However, the extent and underlying resistance mechanisms remain poorly understood [53–55]. The lack of standardized cultivation and phenotyping protocols [50] and the genome plasticity, challenging the genome-resolved metagenomics for *Arcobacter* species [51, 56], have delayed the description of genotypic and phenotypic resistance features of *Arcobacter* species. Although requiring further phenotypic confirmation, the latent, putatively mobile ARG characterized here aids in elucidating previously unrecognized resistance features of *Arcobacter* species.

Gene *bla*MCA may represent another putative resistance feature that has yet to be widely recognized. To our best knowledge, this gene has been referred to only once before [39]. Its absence in most of the widely used ARG databases, such as ResFinder [57] and CARD [58], and therefore scarce representation in the literature, would suggest that *bla*MCA is lacking functional verification and considered rather an intrinsic than acquired ARG. However, observing identical copies of *bla*MCA within p*dif*-modules among highly varying genetic regions here could be indicative of relatively recent gene acquisition events, possibly via p*dif*-site mediated ARG decontextualization [43]. Our findings of *bla*MCA p*dif*-modules coupled with IS elements may refer to additional mobility potential or transcription of *bla*MCA [59]. Understanding the significance of the p*dif*-sites as ARG mobilizers has been impeded by the difficulty in identifying them [39] as well as restricted focus on well-established ARG-modules and human pathogens [39, 60]. Here we described p*dif*-modules and their associated ARGs and hosts at a microbial community level, without limiting the analysis to plasmids [39] or specific species [61].

*Acinetobacter* sp. was the only *bla*MCA host resolved here. In contrary to prior knowledge [39] and all other contigs in our analysis, in this particular case, the p*dif*-module seemed to be located within a chromosomal rather than plasmid context. Chromosome-integrated p*dif*-modules have been reported before [61, 62], and suggested to play roles similar to those of plasmid p*dif*-modules [61]. While particularly common in *Acinetobacter* plasmids [43], ARG encoding p*dif*-modules have been detected across genera [39], confirming that p*dif*-module-mediated spread of resistance features across bacterial lineages can occur, and environments like wastewater could host conditions enabling such cross-species transmission. Both latent ARG hosts, *Arcobacter* and *Acinetobacter*, belong to genera comprising opportunistic pathogens and environmental lineages, and are widespread across environmental and human- and animal-associated sources, including wastewater [48]. Given the increased likelihood of HGT between taxonomically related and environmentally co-occurring species [63], these taxa may play an elevated role as mediators of ARG transfer between non-pathogenic and pathogenic species [12, 15].

### Wastewater resident taxa as the carriers of established, clinically significant ARGs

In addition to the latent ARGs, our methylation-based binning approach identified previously unreported environmental bacteria hosts for clinically relevant ARGs. *erm*(F) gene is among the most commonly reported ARGs in wastewater [49, 64–66], particularly sludge [67] and anaerobic digestion systems [68]. Some studies even report its enrichment as a result of the activated sludge process [66, 69]. However, the underlying factors, particularly bacterial host dynamics related to this, are still not well understood. Our findings hinted that elevated *erm*(F) levels in sludge might be driven by multiple host taxa rather than proliferation of one species. Based on our observations on *erm*(F) carriers detected by distinct methylation profiles, the host range seemed to have diversified during wastewater treatment: In accordance with the marked abundance of gut‑associated Bacteroidales in human gut, they are also common in influent wastewater [70, 71] and unsurprisingly identified as *erm*(F) hosts in influent also in this study. Instead, a total of 12 *erm*(F) genome bins representing six environmental Bacteroidales families were recovered from dried sludge. Partially overlapping Bacteroidales families have been described as key taxa in activated sludge and anaerobic digestion communities, where they contribute to amino acid degradation [67, 70, 72]. Along with the broad host range of *erm*(F), the diverse genetic contexts across and within Bacteroidales species could point to relatively recent acquisition events. Although the ARG mobilizing mechanisms are well described for gut associated Bacteroides [71, 73–75], further study is needed to better understand MGEs of environmental Bacteroidales involved in ARG trafficking. Given that dried sludge is commonly further processed into soil products, the diverse Bacteroidales in sludge could serve as reservoirs for *erm*(F), potentially facilitating their spread or accumulation in both human gut-associated and environmental Bacteroidales species.

Other clinically relevant integron-encoded ARGs found in strictly environmental species, such as *bla*OXA-129 in wastewater denitrifier *Simplicispira* [52] and *sul1-9* in *JAUCQB01* sp. of phylum Planctomycetota (class Phycisphaerae) [76] provided further support on the contribution of environmental bacteria or wastewater resident taxa to the overall resistance burden and their role as putative intermediate hosts and mobilizers of integron-encoded ARGs in wastewater [2, 7].

### Evaluation of the methylation-based binning method

By linking ARGs to their broader genetic contexts and hosts at the microbial community level, this study addresses one of the key challenges in the field hindering the environmental resistome risk assessment [2, 15]. Another recent approach to this task is high‑throughput single‑cell metagenomic sequencing, in which individual cells are uniquely barcoded prior to sequencing [77]. While promising, this approach has certain limitations, including the laborious sample preparation, dependency on specific reagents and equipment, and possible bias against gram-negative bacteria [77]. Another, strictly computational strategy for coupling taxonomy, ARGs, and their genetic context draws upon alignment-free methods with CARD k-mers [78]. However, the main weakness of this method is its reliance on established ARGs in a certain database and the assumption that these ARGs occur in expected context sequences, and especially among pathogens, while latent ARGs and any ARGs within new genetic contexts are missed. In contrast, here, we demonstrate that DNA methylation signals, combined with long‑read sequencing, can be used to resolve the bacterial and genetic backgrounds of both established and previously uncharacterized latent ARGs without the dependency of prior knowledge.

Even though the genome bins curated from wastewater represented predominantly partial genomes rather than fully complete MAGs, they provided sufficient context sequence information for ARG mobility potential evaluation and confident taxonomic classification, thereby achieving the central objective of this study. The concordance between the GC-contents of the formed genome bins and the reference genomes of the assigned taxa supported the validity of our findings. Furthermore, consistent ARG-host linkages were recovered from two separate samples (*bla*OXA-129 carried by *Simplicispira* sp. and *sul1-9* carried by *JAUCQB01* sp.), making it highly unlikely that methylation-based linkages were erroneous in both cases.

Building on the valuable work carried out on simpler communities [20, 21], our results demonstrate that methylation signals can be used to resolve plasmid origins also in highly complex communities, yet with critical limitations. Although we could assign the bacterial hosts for some, likely narrow-host range plasmids, unfortunately, none of them represented ARG-encoding plasmids, which are typically conjugative or mobilizable and thus broad‑host‑range plasmids [79]. We speculate that this would be due to the co-occurrence of multiple hosts carrying similar plasmids within the highly complex wastewater microbiome. This might confuse the methylation signal detection, as reads coming from different strains and hence with mixed methylation signatures would be aligned to the same sequence. Horizontally acquired genetic material may give rise to heterogenous methylation profiles due to marks of their past hosts [34]. However, this effect is expected to be transient, becoming diluted or overwritten by host methyltransferases during replication, as unmethylated motifs are targeted by restriction–modification systems [21, 80], and thus would not substantially affect the methylation-based binning strategy. Exception to this can arise when the incoming element, for instance conjugative plasmid or bacteriophage [80–82], encode its own methyltransferase [80], other anti-restriction genes or mobile restriction–modification modules [83]. For biological interpretation of ARG carriage in the framework of this work, distinguishing between hosts at the time of sampling and in recent past has limited relevance. Still as the broad‑host‑range plasmids are key drivers of ARG dissemination [84], further knowledge on their methylation profile dynamics is required for improved methylation-based binning.

Along with the increasing community complexity, methylation patterns become less unique across genomes, making it more difficult to distinguish them [21]. Consequently, the high number of contigs with inconclusive methylation patterns, and the resulting loss of data for downstream genome binning remained as the main limitation of this study, as it substantially reduced the number of ARG-host linkages that could be resolved from the wastewater samples. Future advances in base modification detection sensitivity [85, 86] are expected to enhance methylome resolution. Overall, the limitations of methylation‑based binning differ from those of traditional methods that rely on sequence coverage and composition [18, 87]. For instance, our approach showed indications of strain‑level resolution by recovering distinct genome bins within the same Bacteroidales species. Species-specific methylation profiles of Bacteroidales, particularly *Bacteroides*, have been reported before, although in less diverse mouse gut microbiomes [21]. This may reflect both high methylation profile specificity within this group and the ability of our approach to resolve them in complex communities. Altogether, the application of the presented methylation-based binning approach may be particularly well suited for less diverse samples, or in combination to other binning tools to mitigate the weaknesses of both approaches.

## Conclusions

By the introduction of a novel methylation-based workflow applied to re-connect metagenomic contigs, this work sheds light on the previously hidden hosts of established and latent ARGs in wastewater microbiomes. Our results highlight the importance of culture-independent investigations on resistance features and their carrier bacteria beyond the few clinically most important pathogenic species. Further studies are needed to assess the broader occurrence of the latent beta‑lactamase genes described here and to validate their resistance phenotypes, as current evidence is based solely on computational predictions.

## Methods

### Sample collection and DNA extraction

Detailed sample descriptions of the wastewater metagenomes (PRJEB106782 [88], PRJEB83306 [89]) investigated here are provided in our previous work [11]. Briefly, composite samples were collected from influent (INF1, INF2, INF3), effluent (EFF1, EFF2, EFF3), and dried sludge (SLU1, SLU2, SLU3) at one of the two wastewater treatment plants responsible for processing wastewater in the Helsinki metropolitan area in Finland during three days in 2019 (18.3.2019, 20.3.2019, 26.3.2019). The samples were transported into the laboratory within two hours after collection and processed immediately (including filtration through a 0.22 µm membrane for water samples) and stored in −80 ºC until DNA extraction. For wastewater samples, DNA was extracted from filters using the Qiagen DNeasy PowerWater kit with bead beating according to the manufacturer’s instructions. DNA from dried sludge samples was extracted using the DNeasy PowerSoil kit. The extracted DNA was stored in −20 ºC prior sequencing. Negative controls were not included in this study. As contamination is generally of greater concern in low-biomass microbiome studies [90], we prioritized sequencing depth to maximize ARG recovery from our high-biomass wastewater samples. To minimize contamination risk, filtration units were covered during processing and all instruments were autoclaved.

Synthetic community data (PRJEB85727) [91] of the study by Partanen and colleagues [33] and associated genome sequence data (PRJNA1047486) [92] by Hogle and colleagues [93] were utilized here as the validation data for the correctly reconnected metagenomic contigs. Given a priori taxonomic assignments of the metagenomic contigs, we assessed the applicability of methylation signatures for separating contigs according to their origin. The synthetic communities comprised of 36 species [33], of which 18 isolate whole genome sequences (WGS) were available in public sequence data repository [93]. A comprehensive description of the synthetic communities is provided in the work by Partanen [33] and Hogle and colleagues [93] and Additional file 3: Supplementary Methods.

### Sequencing, HiFi read generation, and metagenomic assembly

Library preparation and PacBio Single Molecule Real-Time (SMRT) sequencing for metagenomic long-reads of wastewater communities were performed at the Institute of Biotechnology, University of Helsinki. Detailed Snakemake (v7.17.1) [94] workflows for the read‑preparation analyses are described in the project GitHub repository [95]. Briefly, highly accurate long-reads (HiFi reads) of metagenomic community samples with kinetic tags were generated from the subreads using PacBio BAM toolkit programs (https://github.com/PacificBiosciences/pbtk) to allow methylation detection in downstream analyses (workflows “Snakefile_HAMBI_preanalysis” and “Snakefile_WW_HiFi_reads” for synthetic and wastewater community data, respectively). HiFi reads without kinetic information (workflows “Snakefile_HAMBI_preanalysis” and “Snakefile_WW_HiFi_reads_no_kinetic_tags” for synthetic and wastewater community data, respectively) were employed for metagenomic assembly. Each of the synthetic and wastewater community samples (five and nine samples, respectively) was assembled separately using hifiasm-meta (v.0.18.0) [96] with the default options.

### Preliminary methylation detection analyses

Next, the kinetic tags recorded during sequencing were summarized as base modifications at the contig level (workflows “Snakefile_HAMBI_preanalysis” and “Snakefile_WW_preanalysis” for synthetic and wastewater communities, respectively). Briefly, HiFi reads with attached kinetic tags were aligned to assembled metagenomic contigs using the PacBio program pbmm2 (v1.10) (https://github.com/PacificBiosciences/pbmm2/). Base modifications were identified using the ipdSummary program (v.3.0) [97] program by comparing observed interpulse duration (IPD) values at each aligned sequence position with those expected for unmodified sequences. Statistically significant comparisons were reported by the program as rows in contig‑specific GFF output files, and base modifications were identified as types of m6A, m4C, or, when unclassified, as “modified base”. Along with the contig name and modification type, the position of the methylated base and its flanking region of 20 bp upstream and downstream were recorded for the following analysis steps.

### Methylation data conversion to Position Weight Matrices (PWMs)

The obtained contig-wise summaries of the detected methylated bases were converted into Position Weight Matrices (PWM) to be used as methylation signatures for further analysis. For that, in-house Python scripts (“scoring_matrices_HAMBI.py”, “scoring_matrices_WW.py” for synthetic and wastewater communities, respectively) were used to extract the flanking sequences separately for each modification types and samples (wastewater samples), and then to convert those data into PWMs. In the PWM files, each row represented one nucleotide (A, T, G, or C) and a column indicated each position surrounding the methylated base [− 20, 20]. This sequence length was chosen based on the previously applied thresholds for de novo methylation motif prediction for instance by the algorithm of MultiMotiMaker [31] tool. Moreover, by retaining the full 41 bp region reported by ipdSummary [97] program, we aimed to enhance the resolution of downstream methylation analysis by preserving sufficient flanking sequence surrounding the modified bases. More detailed description of the constructed PWMs is provided in the Additional file 3: Supplementary Methods.

### Assigning taxonomical labels for the metagenomic contigs of the synthetic communities

To assign taxonomic and genetic element labels (plasmid or chromosome) for the metagenomic contigs from the synthetic community, the assemblies were queried against whole‑genome sequencing (WGS) data from the 18 strains using BLAST [98]. The BLASTn results were parsed by filtering the hits by > 1000 bp and adding the columns of all taxonomic levels (from domain to species) and genetic element (plasmid or chromosome). Those with no hits were set as “NA”.

### Visualization of PWMs and methylation-based binning for the synthetic community

UMAP (Uniform Manifold Approximation and Projection) [99] was applied to visualize and reduce the complexity of the high-dimensional data by projecting it into two dimensions. By the non-linear dimensionality reduction technique of UMAP, known for its ability to preserve both local and global structures in data [99], this approach allowed us to explore the clusters represented by methylation profiles. As a method validation analysis, the a priori known taxonomic identities of metagenomic contigs from the synthetic community were color‑coded to facilitate evaluation of taxon‑wise clustering based on PWMs and visualized using UMAP in an interactive Plotly scatter plot [100]. Additionally, information on the square root transformed sequence lengths computed by SeqKit (v2.5.1) [101], was added to the UMAP visualization. In addition to visual inspection of the clustering of species-wise colored contigs in UMAP, contigs clustering together were gathered and combined into (sample-wise) genome bins. The completeness and redundancy measures were implemented by running CheckM2 (v 1.0.1) [32] to evaluate the performance of the methylation-based binning approach. GTDB-Tk (v2.4.1, database release 220) [102] was used for the taxonomical identity assignment of the genome bins.

As an additional visualization of the generated methylation signatures, sequence logos [103] displaying nucleotide information content were used to assess similarities in base preferences surrounding the modified base across contig clusters (Additional file 2: Fig. S1).

### Identification of established and latent ARG-carrying genome bins in wastewater

The visualization of PWMs was performed similarly to the synthetic community described above. Contig clusters projected in UMAP were examined by visual inspection for each sample separately aiming for subsequent formation of genome bins. The large cluster of indistinguishable data points observed in each sample was excluded from the main analysis to allow a more accurate assessment of data points where distinct clustering patterns were evident (Additional file 2: Fig. S2). The excluded data represented contigs showing inconclusive methylation data, restricting their eligibility by our approach. The number of contigs that were included in the final analysis following data exclusion is summarized in Table 2. Subsequently, a magnified view of the clusters in the interactive scatter plot was used to enable detailed inspection and identification of clustered contigs.

Established and latent ARGs present in the contigs were predicted using ResFinder (v 4.7.0) [57] and fARGene (Fragmented Antibiotic Resistance Gene iENntifiEr) (v0.1) [40], respectively. By color-coding the contigs with ARGs in the UMAP visualization, we directed the focus on the formation of genome bins with ARGs. The need for manual processing steps in the methylation-based binning approach restricted the volume of data that could be examined for ARG-context or -host linking. This was particularly highlighted for the numerous latent ARGs detected by fARGene, with which we decided to focus on the putative beta-lactamases due to their higher likelihood of serving the antibiotic resistance function and being mobile in contrast to chromosomal housekeeping genes that are incorrectly assigned as putative resistance genes [5]. Quality metrics and taxonomical identities of the formed ARG carrying genome bins were evaluated similarly as described for the synthetic community above.

### Analysis of ARG mobility potential and genetic context

Five genome bins with established or latent ARGs were investigated in more detail. They were selected as example cases representing newly resolved ARG-host linkages or understudied potentially emerging resistance features. Additionally, those ARGs that were detected within different contig clusters suggesting multiple ARG hosts in wastewater were prioritized (Additional file 2: Fig. S4).

Detailed analysis scripts for the ARG genetic context analyses are provided in the project GitHub page (“WW_analyses/Genetic_context_analyses.md”) [95]. In addition to the ARGs present in the binned contigs, hits to the same ARGs were queried against all wastewater data using BLAST [98]. ARG flanking regions (5 or 10 kb upstream and downstream) of both, binned contigs from the methylation approach and unbinned contigs lacking distinctive methylation data, were extracted using in‑house SeqKit‑based (v 2.5.1) [101] scripts (“extract_regions.sh” in the project GitHub page [95]). Bakta (v1.11.0) [104] was used for gene annotation in the flanking regions. Plasmid and p*dif*-site predictions for the entire contigs or extracted ARG flanking regions, respectively, were performed using geNomad (v1.8.0) [105] and pdifFinder [39]. To examine the diversity among the ARG genetic contexts and to reduce the number of sequences for the visualization, the genetic contexts were dereplicated to exclude identical or similar genetic contexts by vsearch (v2.22.1) [106]. After removing redundancy, the remaining sequences were visualized using pyGenomeViz (v1.6.1) [107] using a BLAST [98] query for highlighting the aligned nucleotide sequences between the compared sequences. The insertion sequences located near the investigated ARGs were examined in more detail by the online server of ISfinder [108], while the *attC* sites were detected by HattCI program [109].

Of the five examined ARG genome bins, ARG gene sequences were extracted for phylogenetic analysis for all except *bla*OXA-129 and *sul1-9* sequences, which were few and identical or nearly identical. Gene alignment was done using MAFFT (v7.505) [110], for which the tree was computed by RAxML (command raxmlHPC-PTHREADS) (v8.2.12) [111] and visualized with Interactive Tree of Life (iTOL) [112]. The phylogenetic trees of the ARGs were visualized along with the genetic contexts. Further details regarding the ARG context analyses are found in the Additional file 3: Supplementary Methods.

### Taxonomical composition of wastewater communities

Relative abundance of genus-level taxa in wastewater communities was analyzed using Sylph (sylph (v 0.8.1) and sylph-tax (v 1.5.1)), with metagenomic reads as the input [113]. Genera with relative abundance below 0.5% were left out of the visualization.

### Threshold selection for methylation input data using a Random Forest classifier on synthetic community data

Before proceeding to the methylation-based genome binning of wastewater samples, preliminary analyses were conducted to assess how the amount of modification input data influences downstream analyses and thereby inform threshold selection. For this, a Random Forest model was implemented and trained with default parameters with Scikit-learn (v1.4.2) [114] Python library on the PWMs and a priori known taxonomical labels of the synthetic community data. This included the construction of 100 decision trees, each trained using bootstrap sampling, with the number of features considered at each node set to the square root of the total number of features. The input data set was split into 80% and 20% training and testing data sets, respectively, and stratified for each species in order to predict the taxonomic labels of the metagenomic contigs using the high-dimensional methylation matrix. Model performance was evaluated using a confusion matrix, which summarizes the number of correct and incorrect predictions (Additional file 2: Fig. S9). Overall accuracy was calculated as the proportion of correctly classified instances to the total number of instances (Additional file 2: Fig. S9). The prediction accuracy was tested using different thresholds for modification input data 20, 50, 100, 200, and 500 (Additional file 1: Table S11).

While classifier accuracy increased with more stringent threshold values, this improvement was accompanied by a concurrent reduction in the number of detectable species (Additional file 1: Table S11). This suggested that the decreased amount of input data may have led the model to fit noise in the training data, thereby limiting its generalizability to unseen samples. Of the evaluated thresholds, a cutoff of > 50 modifications per contig achieved the highest model accuracy (0.88) while retaining detection across diverse species labels (Additional file 1: Table S11). Although with increasingly stringent cutoffs fewer contigs are available for the binning and, consequently, less complete bins or MAGs can be formed. However, given the several‑fold higher volume of methylation information expected for the extensively diverse wastewater communities compared with the synthetic communities, the recognition of unique methylation profiles as distinct contig clusters in UMAP visualization was anticipated to be more challenging. Therefore, a threshold of 50 was selected to balance noise reduction (arising from indistinguishable methylation data points) with the retention of a sufficient amount of data for downstream analyses, enabling the successful curation of high‑quality ARG‑context and -host linkages from contig clusters identified in UMAP visualizations (Fig. 2, Additional file 1: Table S2). Particularly, with wastewater samples, we did not aim for high quality MAGs but rather to connect as many contigs together as possible and what was required for ARG host identification. These considerations supported our choice of a more conservative threshold for complex wastewater communities, whereas lower thresholds may be preferable for simpler microbial communities to retain a larger number of contigs for more complete genome binning.

Overall, the Random Forest classifier predicted synthetic community members with high accuracy (Additional file 2: Fig. S9), supporting the selected target (methylation signatures), their presentation (PWMs), and overall approach. However, species‑level variation was also noted. For instance, *Cupriavidus oxalaticus* and *Comamonas testostorini_C* showed low prediction accuracies (Additional file 2: Fig. S9), suggesting misclassifications across many different species, rather than repeated specific incorrect predictions, suggesting rather heterogeneous and inconclusive methylation profiles instead of substantially overlapping profiles between multiple species. However, because these contigs were located within the miscellaneous group in the UMAP visualization and were clearly separated from the well‑defined species‑level clusters, the risk of false positives arising from such misclassifications is mitigated by downstream analysis steps.

### Further details

All other figures that were not specifically mentioned above were drawn using Plotly [100] and RStudio (2024.12.1 + 563.pro5) [115] by applying packages phyloseq (v1.16.2) [116], ggplot2 (v3.5.0) [117], and vegan (v2.6–10) [118]. Comprehensive descriptions of the materials and methods can be found in the Additional file 3: Supplementary Methods.

## Supplementary Information

Additional file 1: Supplementary Tables S1-S11.

Additional file 2: Supplementary Figures S1-S9 [113, 118, 121–123].

Additional file 3: Supplementary Methods [32, 33, 40, 93, 97, 98, 100–104, 106, 107, 110–112, 114].

## Data Availability

The subread sequence data from the wastewater metagenomes are available in the European Nucleotide Archive (ENA) under accession number PRJEB106782 [88], along with the corresponding HiFi reads, which can be found under accession number PRJEB83306 [89]. The other sequencing data used in this study were accessed via the following accession numbers: PRJEB85727 [91] for the synthetic community metagenomes [33] and PRJNA1047486 for the whole genome sequence data [92] of the 18 strains listed in Additional file 1: Table S1 of the synthetic communities [93]. The genome bin sequences are available in Zenodo repository [119]. The analysis scripts are shared in the publicly available GitHub [95] and Zenodo repositories [120] under the GNU General Public License v3.0 (GPL-3.0) license.
