# Supplementary material for: Long-read metagenomics and methylation-based binning support the discovery of antibiotic resistance gene–host associations in complex communities
Source: Genome Biol. 2026 Jul 13;27:306. doi: 10.1186/s13059-026-04200-0 (PMC13644095; doi:10.1186/s13059-026-04200-0)
Supplement: Supplementary file 1 [file 13059_2026_4200_MOESM2_ESM.docx]

**Long-read metagenomics and methylation-based binning support the discovery of antibiotic resistance gene–host associations in complex communities**

Melina Markkanen^1,2^*, Heidi Putkuri^3^, Dovydas Kičiatovas^2,3^, Ville Mustonen^2,3^, Marko Virta^1,2^, Antti Karkman^1,2^*

^1^Department of Microbiology, University of Helsinki, Helsinki, Finland

^2^Multidisciplinary Center of Excellence in Antimicrobial Resistance Research, University of

Helsinki, Helsinki, Finland

^3^Department of Computer Science, Department of Organismal and Evolutionary Biology, University of Helsinki, Helsinki, Finland

*Corresponding authors: [melina.markkanen@helsinki.fi](mailto:melina.markkanen@helsinki.fi), [antti.karkman@helsinki.fi](mailto:antti.karkman@helsinki.fi)

List of Supplementary Figures:

**Fig. S1.** Sequence logos of synthetic community

**Fig. S2.** UMAP ordination of methylomes of wastewater communities

**Fig. S3.** Bacterial composition of the wastewater community

**Fig. S4.** *erm*(F) genome bins in different wastewater samples

**Fig. S5.** PCoA of bacterial composition in wastewater

**Fig. S6.** Composition of the Bacteroidales order in wastewater communities

**Fig. S7.** Genetic contexts of *bla*OXA-129

**Fig. S8.** Genetic contexts of *sul1-9*

**Fig. S9.** Random Forest classifier predictions

**Supplementary Results**

*Aeromonas caviae* (chrom)

*Sphingobacterium spiritivorum* (chrom)

*Pseudomonas_E chlororaphis* (chrom)

*Azospirillum brasilense_B*

*Azorhizobium caulinodans*

*Paracoccus denitrificans* (chrom, plas)

*Sphingobium yanoikuyae* (chrom, plas2)

*Agrobacterium tumefaciens* (chrom2, plas)

*Morganella morganii* (chrom)

**Fig. S1.** Sequence logos of contigs included in synthetic community bins and MAGs (**Fig. 1**, Additional file 1: **Table S2**). Sequence logos of two example contigs are shown. The examples were selected based on the following criteria: contigs from separate samples and different genetic elements, if applicable (chromosomes (chrom1-2) or plasmids (plas1-2)).

**A.	B. C.**

**D. E. F.**

**G. H. I.**

**Fig. S2.** UMAP ordination of methylomes of wastewater communities. The heatmap color encoding denotes the number of detected base modifications per contig. A logarithmic transformation was applied to the modification count data to reduce skewness. The dot size indicates the contig length, which is square root transformed. In **A-F**, the included data is shown with a rectangle with a dotted line, while in **G-I**, all data except those contigs within the cluster a marked with a red solid rectangle.

**Fig. S3.** Bacterial composition of wastewater communities. Genera with less than 0.5 % relative abundance computed by Sylph (Shaw et al. 2025) were filtered out from the visualization. Phylum-level taxa are shown.

**Fig. S4.** *erm*(F) bins in different wastewater samples. The plot on the top row represents influent sample INF3, while the bottom row plots show the UMAP visualization of the metagenomic contigs of sludge samples SLU1, SLU2, and SLU3. In all plots, the pure red colored data points denote contigs with the *erm*(F) gene. The other dots (pale yellow or pink) represent non-*erm*(F) contigs. Contigs clustering together with each *erm*(F) contigs were combined into bins, and the labels of the bins are displayed on the plots.

|  | Df | Sumofsqs | R2 | F | Pr(>F) |
| --- | --- | --- | --- | --- | --- |
| Model | 2 | 2.05630 | 0.92591 | 37.493 | 0.005 ** |
| Residual | 6 | 0.16453 | 0.07409 |  |  |
| Total | 8 | 2.22083 | 1.00000 |  |  |

**Fig. S5.** Principal Coordinates Analysis (PCoA) of bacterial composition in wastewater. Ordination was plotted with genus-level relative abundances generated by Sylph (Shaw *et al.* 2025) using Bray-Curtis dissimilarity by the vegan package (Oksanen *et al.* 2020). Microbial community composition differed significantly between the sample types (p = 0.005, highly significant (p < 0.01)). F = 37.493: A high F-statistic suggested strong separation between groups. About 92.6% of the variation in Bray-Curtis distances is explained by sample type (Permutation test for adonis under reduced model, Permutation: free, Number of permutations: 999).

**Fig. S6.** Composition of the Bacteroidales order in wastewater communities computed with Sylph (Shaw et al. 2025). Family-level taxa are shown.

**Identification of newly recognized hosts for integron‑encoded established ARGs**

Two genome bins assigned to *Simplicispira* sp. (INF1-C3, INF3-C5) were identified as newly described *bla*OXA-129 gene carriers in influent wastewater by two separate samples. Comparative genomics analysis on the *bla*OXA-129 carrying *Simplicispira* sp. genome bins, unbinned sequences from the wastewater and previously described references (*Enterobacter hormaechei* (FJWZ01000025) and *Pseudomonas aeruginosa* (CP031449.2)) (Valiatti *et al.* 2024; Liu *et al.* 2010) revealed both shared and unique features of the integron cassette arrays and the *bla*OXA-129 flanking region within different hosts (**Fig. S7**, Additional file 1: **Table S9**). The integrases of *Simplicispira* sp. were dissimilar to those in *E. hormaechei* and *P. aeruginosa* which in turn resembled each other (**Fig. S7**). However, according to BLAST search against public sequence databases, similar integron integrase genes to those in *Simplicispira* sp. are found in various other taxa including *Klebsiella* and *Acidovorax* (data not shown). While aminoglycoside ARG *aadA* was found in the integron cassette arrays of both *E.* *hormaechei* and *Simplicispira* sp., the remaining numerous *attC* site flanked gene cassettes in the array of the latter host contained genes encoding proteins of unknown function. Another notable difference between the context regions of the clinical *E. hormaechei* and of *P. aeruginosa* and those in *Simplicispira* sp. was the presence of IS6 family IS6100 transposases flanking the integron cassette in the former and their absence in the latter, pointing to a greater potential for mobilization of the cassettes in the reference strains (Johansson *et al.* 2023) (**Fig. S7**).

**Fig. S7.** Genetic contexts (10 Kb upstream and downstream) of *bla*OXA-129 (shown in red) in the methylation-based genome bins (*Simplicispira* sp.) and unbinned contigs. Sequences of previously described *bla*OXA-129 encoding strains of *Enterobacter hormaechei* (FJWZ01000025.1) and *Pseudomonas* *aeruginosa* (CP031449.2) are included as references.

High-quality genome bins of *JAUCQB01* sp. (class Phycisphaerae) carrying *sul1-9* (SLU2-C4, SLU3-C1) were recovered from two separate sludge samples. Variant *sul1-9* differs from its closest relative *sul1-2* by an 11 bp long tail and one nucleotide substitution. Exploring the *sul1-9* contexts of the sequences of this study and of the previously reported hosts of *Escherichia coli* (CP026207.1) and *Salmonella enterica* (AY963803.6) revealed a shared integron gene array structure for all the examined sequences (**Fig. S8**, Additional file 1: **Table S10**). Moreover, similarly to the *bla*OXA-129 encoding integron gene cassettes, IS6 family IS6100 transposase genes were detected in all investigated sequences including both reference sequences from public databases as well as the newly described *sul1-9* hosts of *JAUCQB01* sp. and unbinned contig characterized in this study (**Fig. S8**). In contrast to the *E. coli* and *S. enterica* reference strains, no additional ARGs were present in the integron cassettes of this study (**Fig. S8**). However, unlike for the reference strains, no *attC* sites were reported for the sequences of this study (**Fig. S8**).

**Fig. S8** Genetic contexts of *sul1-9* (shown in red) in the genome bins (*JAUCQB01* sp., class Phycisphaerae) resolved by methylation-based binning and unbinned contigs with no resolved taxonomical identity. *Escherichia coli* (CP026207.1), and *Salmonella enterica* (AY963803.6) encoding *sul1-9* are included as reference strains for the comparative genetic analysis of 10 Kb upstream and downstream flanking region of *sul1-9* gene.

**A. B.**

**Fig. S9.** Random Forest classifier predictions for the taxonomic labels of the metagenomic contigs using the high-dimensional methylation matrix. Predictions are shown for **A.** Train Set and **B.** Test Set.
