# Supplementary material for: Long-read metagenomics and methylation-based binning support the discovery of antibiotic resistance gene–host associations in complex communities
Source: Genome Biol. 2026 Jul 13;27:306. doi: 10.1186/s13059-026-04200-0 (PMC13644095; doi:10.1186/s13059-026-04200-0)
Supplement: Supplementary file 2 [file 13059_2026_4200_MOESM3_ESM.docx]

**Long-read metagenomics and methylation-based binning support the discovery of antibiotic resistance gene–host associations in complex communities**

Melina Markkanen^1,2^*, Heidi Putkuri^3^, Dovydas Kičiatovas^2,3^, Ville Mustonen^2,3^, Marko Virta^1,2^, Antti Karkman^1,2^*

^1^Department of Microbiology, University of Helsinki, Helsinki, Finland

^2^Multidisciplinary Center of Excellence in Antimicrobial Resistance Research, University of

Helsinki, Helsinki, Finland

^3^Department of Computer Science, Department of Organismal and Evolutionary Biology, University of Helsinki, Helsinki, Finland

*Corresponding authors: [melina.markkanen@helsinki.fi](mailto:melina.markkanen@helsinki.fi), [antti.karkman@helsinki.fi](mailto:antti.karkman@helsinki.fi)

**Synthetic community samples**

The full sample preparation and experimental setup of the synthetic communities used here for the methylation method validation are detailed in Partanen *et al.* 2025. Briefly, synthetic communities were composed of 36 different bacterial strains and grown in microcosm bottles for 20 days with maintenance every 96 hours by the addition of 2.5 % of the fresh community Partanen *et al.* 2025. The community samples were subjected to treatments involving one of five antibiotics and varying temperatures, and their combinations altogether in five replicates, reflecting the research aims of the study by Partanen and colleagues (2025). Among the 75 microcosm experiments, five individual samples were included in this work to serve as a validation for the methylation-based method. These samples were bcAd1023T (0.2 µg/ml tetracycline, 15 °C; tc_0.2_15_5), bcAd1037T (20 µg/ml sulphamethazine, 25 °C; su_20_25_3), bcAd1039T (20 µg/ml sulphamethazine, 25 °C; su_20_25_4), bcAd1046T (0.02 µg/ml tetracycline, 37 °C; tc_0.02_37_3), bcAd1063T (no antibiotic, 37 °C; no_0_37_3). Since the experimental treatments did not play a critical role in our analyses, the samples were randomly selected. According to Partanen *et al.* 2025, the sequencing was performed by using PacBio instrument Revio.

The available whole genome sequence data of the Partanen *et al.* 2025 community members published by Hogle *et al.* 2023 (<https://gitlab.utu.fi/slhogl/hambiLongRead>) was retrieved for the method validation purposes. Because of the experimental research design in the study by Partanen *et al.* 2025 this meant WGS data for half (18/36) of the species present in our method validation communities. The sequencing of the community members by Hogle *et al.* 2023 was performed before setting up the experiments by Partanen *et al.* 2025. Similarly to the wastewater metagenomes here, the WGS data by Hogle *et al.* 2023 was generated by PacBio Sequel II.

**Methylation data conversion to Position Weight Matrices (PWMs)**

The values in the PWMs represented relative frequencies of nucleotides, normalized against a background model to account for baseline nucleotide frequencies in the contig. The normalized relative frequency $S_{i}^{c}\left( a \right)$ for each nucleotide $a$ at each position $i$ (where $i \in\left[ -20,20 \right]$) within the context sequences for contig $c$ is calculated using the following formula:

$$S_{i}^{c}\left( a \right) = \log\frac{q_{i}^{c}\left( a \right)}{p^{c}\left( a \right)}$$

The term $q_{i}^{c}\left( a \right)$ represents the relative frequency of nucleotide $a$ at position $i$in the contig $c$ calculated using:

$$q_{i}^{c}\left( a \right) = \frac{N_{i}^{c}\left( a \right)+p^{c}\left( a \right)}{s+1}$$

Here, $N_{i}^{c}\left( a \right)$ is the count of nucleotide $a$ at position $i$ in the context sequences, and $s$ is the total number of bases at that position across all context sequences. The $p^{c}\left( a \right)$ is the frequency of the nucleotide $a$. By adding a pseudo count to the denominator, we avoid zero values, which would otherwise lead to undefined or infinite results when computing relative frequencies. This also helps to normalize the contributions of observed counts and background frequencies. As a result, we obtained matrices of 4 x 41 for the three modification types, allowing the analysis of methylated nucleotide positional preferences relative to their distribution throughout the contig. To minimize noise, the methylation type with fewer than 50 detected instances. Finally, the matrices were flattened into one-dimensional arrays, and the sample-wise contigs were concatenated into large files according to the three modification types. In the case of synthetic community data, the labels were attached to these files by matching the contigs’ PWM data with their corresponding taxonomic and genetic element (plasmid or chromosome) identity.

**Sequence logos**

To further confirm the similar methylation profiles of clustered contigs, sequence logos were created to visualize the nucleotide preferences across the context sequences at each position (Schneider & Stephens, 1990). Sequence logos show the distribution of the four bases at each position within the sequence, aiding in understanding the nucleotide preferences by illustrating the relative frequency and importance of each base (Schneider & Stephens, 1990), in our case, revealing the methylation target motif sequences.

The information content (Schneider & Stephens, 1990) *R_i_* for each position *i* which was calculated using the formula:

$$R_{i} = \log_{2} \left( s \right) - \left( H_{i}+e_{n} \right)$$

where *s* is the number of possible nucleotides, *n* the number of sequences, and *H_i_*

is the uncertainty (or Shannon entropy) at position *i* in the context sequence:

$$H_{i} = -\sum_{b=1}^{t} f_{b,i} \times\log_{2} f_{b,i}$$

and the function *f_b,i_* is the relative frequency of nucleotide *b* in position *i*. The

*e_n_* is the approximation for the small-sample correction calculated with

$$e_{n} = \frac{1}{\text{ln}2} \times\frac{s-1}{2n}$$

In the logo image, the total height of the letters illustrates the information content

of a specific position in the sequences converted into bits:

$$height = f_{b,1} \times R_{i}$$

**Analysis of ARG mobility potential and genetic context**

The latent class C or D beta-lactamases represented by methylation-resolved genome bins were used to query similar unbinned sequences in the full dataset by BLAST (Camacho *et al.* 2009). Bakta (v1.11.0) (Schwengers *et al.* 2021) was used for gene annotations. To extract the ARG sequences from their context, a BLAST comparison between the contigs with matches and the ORF sequences predicted by fARGene was performed to identify the sequence locations. For the analysis of the ARG flanking gene region, this gene location information was used to obtain sequences of 5 or 10 Kb upstream and downstream of the ARG for further analysis by in-house scripts (see detailed analysis steps at <https://github.com/melinamarkkanen/Methylation/blob/main/WW_analyses/Genetic_context_analyses.md>) applying SeqKit tools (v 2.5.1) (Shen *et al.* 2016).

For mobility prediction and detailed gene context analysis, flanking regions were dereplicated for the visualization purposes. For that, vsearch (v2.22.1) (Rognes *et al.* 2016) was run for all binned and unbinned sequences of class D and C beta-lactamases separately using sequence identity thresholds of 0.99 and 0.90 for respectively. For the established gene *erm*(F) the threshold for the dereplication performed by vsearch (v2.22.1) (Rognes *et al.* 2016) was 0.50. After dereplication, all sequences that had been assigned to genome bins but were removed during dereplication were reinstated for the visualization. Additionally, reference sequences that either matched the predicted genes or represented the closest related established ARGs retrieved from public sequence repositories were included in the visualization along with their flanking regions. The final manual data exclusion step was performed by excluding sequences in which genes of interest were truncated by visual inspection.

In addition to *Acinetobacter* sp.*,* (bin INF1-C3f), the latent ARGs of class C beta-lactamases were also found to be hosted by *Aquabacterium_A* sp. (bins INF3_C1f, EFF1-C1f, and EFF2-C1f) and *Acinetobacter cellitus* (bin INF2-C5f) (Additional file 1: **Table S3**). However, the beta-lactamases in the latter two hosts were dissimilar to those identified in *Acinetobacter* sp. As more hits were obtained across the whole data for the gene whose taxonomic identity was assigned as *Acinetobacter* sp., we decided to focus our downstream analyses on this gene.

For the phylogenetic analysis, the gene sequences of the latent beta-lactamases and *erm*(F) genes were oriented consistently (reverse-complemented if needed) for the sequence alignment and gene tree visualization with MAFFT (v7.505) (Katoh *et al.* 2002), RAxML (v8.2.12) (Stamatakis *et al.* 2014) and Interactive Tree of Life (iTOL) (Letunic *et al.* 2021). For the genetic context visualization, GenBank files (.GBFF) obtained from Bakta (v1.11.0) (Schwengers *et al.* 2021) were converted to a consistent orientation by an in-house Python script (see detailed analysis steps at <https://github.com/melinamarkkanen/Methylation/blob/main/WW_analyses/Genetic_context_analyses.md>) if needed before the visualization with pyGenomeViz (v1.6.1) (Shimoyama *et al.* 2024) using a BLAST (Camacho *et al.* 2009) query for highlighting the aligned nucleotide sequences between the compared sequences. The gene colors were set manually according to the Bakta annotation labels.

The analysis for the remaining two established ARGs, *bla*OXA-129 and *sul1-9*, and their contexts was similar to the latent ARGs, except for the dereplication step, which was unnecessary because the number of visualized sequences was sufficiently low. Additionally, no gene trees for the ARGs were drawn as the sequences were identical or differed by only one to two nucleotides from each other.
